# High dietary arachidonic acid levels induce changes in complex lipids and immune-related eicosanoids and increase levels of oxidised metabolites in zebrafish (*Danio rerio*)

**DOI:** 10.1017/S0007114517000903

**Published:** 2017-05-09

**Authors:** Anne-Catrin Adam, Kai K. Lie, Mari Moren, Kaja H. Skjærven

**Affiliations:** National Institute of Nutrition and Seafood Research (NIFES), PO Box 2029 Nordnes, NO-5817 Bergen, Norway

**Keywords:** Metabolomics, Zebrafish, Arachidonic acid, Eicosanoids, Oxidative stress

## Abstract

This study explores the effect of high dietary arachidonic acid (ARA) levels (high ARA) compared with low dietary ARA levels (control) on the general metabolism using zebrafish as the model organism. The fatty acid composition of today’s ‘modern diet’ tends towards higher *n-*6 PUFA levels in relation to *n*-3 PUFA. Low dietary *n-*3:*n-*6 PUFA ratio is a health concern, as *n-*6 PUFA give rise to eicosanoids and PG, which are traditionally considered pro-inflammatory, especially when derived from ARA. Juvenile zebrafish fed a high-ARA diet for 17 d had a lower whole-body *n-*3:*n-*6 PUFA ratio compared with zebrafish fed a low-ARA (control) diet (0·6 in the control group *v*. 0·2 in the high-ARA group). Metabolic profiling revealed altered levels of eicosanoids, PUFA, dicarboxylic acids and complex lipids such as glycerophospholipids and lysophospholipids as the most significant differences compared with the control group. ARA-derived hydroxylated eicosanoids, such as hydroxy-eicosatetraenoic acids, were elevated in response to high-ARA feed. In addition, increased levels of oxidised lipids and amino acids indicated an oxidised environment due to *n-*6 PUFA excess in the fish. To conclude, our results indicate that an ARA-enriched diet induces changes in complex lipids and immune-related eicosanoids and increases levels of oxidised lipids and amino acids, suggesting oxidative stress and lipid peroxidation.

Today’s ‘modern diet’, with an increased consumption of saturated fat, meat and vegetable oil, and a decreased consumption of fish and fresh vegetables, has led to a selective loss of *n-*3 PUFA in favour of *n-*6 PUFA, which results in a decreased *n-*3:*n-*6 PUFA ratio^(^ ^1^ ^,^ ^2^ ^)^. The physiological effects of a decreasing *n-*3:*n-*6 PUFA ratio are diverse, and epidemiological studies indicate that a disproportionally high intake of *n-*6 PUFA may contribute to health problems like the metabolic syndrome, diabetes, obesity, CVD, cancer and other inflammatory, neurodegenerative or autoimmune diseases^(^ ^3^ ^–^ ^8^ ^)^. Generally, high total fat intake increases the risk for health problems according to the 2008 FAO/WHO report^(^ ^9^ ^,^ ^10^ ^)^.

The general view is that the above-mentioned health effects of high-*n-*6 PUFA intake are caused by the potent bioactive metabolic products of PUFA. Essential PUFA, like arachidonic acid (ARA) and EPA are converted to numerous bioactive lipid classes, collectively known as oxylipins (oxidation products of ARA and EPA)^(^ ^11^ ^)^. Oxylipin and ARA levels can be influenced by the diet directly; however, ARA conversion to eicosanoids is a rate-limiting enzymatic process^(^ ^12^ ^)^. Biological functions of those oxylipins, and especially eicosanoids, are traditionally considered anti-inflammatory when derived from *n-*3 PUFA and pro-inflammatory when derived from *n-*6 PUFA. This knowledge evoked focus on the risks and benefits of PUFA consumption^(^ ^13^ ^,^ ^14^ ^)^. On the contrary, Calder^(^ ^15^ ^)^ emphasised that labelling ARA-derived eicosanoids as pro-inflammatory is an oversimplification because of the fact that consumption of *n-*6 PUFA can have variable effects on physiology, with both anti- and pro-inflammatory responses^(^ ^16^ ^,^ ^17^ ^)^. ARA-derived PG (2-series) induce inflammation, inhibit pro-inflammatory leukotrienes and cytokines, and induce anti-inflammatory lipoxins^(^ ^18^ ^,^ ^15^ ^)^.

ARA-derived eicosanoids have been studied intensively, and ARA is widely discussed in the context of signalling cascades regulating inflammation, pain, fever and other homoeostatic actions such as blood pressure, bone metabolism, growth and reproduction^(^ ^19^ ^–^ ^23^ ^)^. These biological functions are traditionally attributed to the immunomodulating lipid mediators such as ARA-derived hydroxy-eicosatetraenoic acids (HETE)^(^ ^24^ ^)^, PG, thromboxanes and leukotrienes^(6,^ ^15^ ^)^. The variety of lipid mediators that regulate physiological functions makes it difficult to elucidate their individual biological roles^(^ ^11^ ^)^. Fatty acids and eicosanoids exert their biological function by changing cell membrane composition and by controlling gene expression through nuclear receptors like PPAR, hepatocyte nuclear factor 4*α* and liver X receptor, and through transcription factors such as NF-*κ*B and sterol-regulatory-element-binding protein^(^ ^13^ ^,^ ^24^ ^–^ ^27^ ^)^. These nuclear receptors are regulated by direct fatty acid- and eicosanoid-binding, or by the regulation of G-protein-linked cell surface receptors, thereby activating signalling cascades^(^ ^24^ ^,^ ^26^ ^–^ ^28^ ^)^.

The zebrafish is an omnivorous, tropical, freshwater fish, and a well-suited model organism for understanding vertebrate metabolism at a molecular and genetic level^(^ ^29^ ^–^ ^32^ ^)^. Knowledge gained from zebrafish studies are highly relevant to both humans^(^ ^33^ ^)^ and fish^(^ ^34^ ^,^ ^35^ ^)^. The focus on the essential fatty acid ARA in fish nutrition is rising, and its impact on health performance and reproduction in both marine and saltwater species is gaining more attention^(^ ^36^ ^–^ ^40^ ^)^. Most of the studies on the nutritional effects of ARA on zebrafish and other fish have focused on stress response, survival, bone development, deformities, reproduction and growth performance^(^ ^22^ ^,^ ^23^ ^,^ ^41^ ^–^ ^43^ ^)^. These studies demonstrate the importance of appropriate dietary ARA levels for maintaining optimal growth, reproduction and overall health, with special emphasis on larval fish requirements^(^ ^36^ ^)^. Powell *et al*.^(^ ^44^ ^)^ investigated the role of ARA and different dietary *n-*3:*n-*6 PUFA ratios in inflammation in zebrafish. They measured key inflammatory markers, growth and body fat in adult zebrafish in response to dietary *n-*3:*n-*6 PUFA ratios. These results indicate that a low *n-*3:*n-*6 PUFA ratio can impact health through metabolic changes when high levels of ARA are provided through diet.

In the present study, we fed zebrafish a diet high or low in ARA. We elucidated the metabolic changes in zebrafish induced by a dietary shift in PUFA composition. The dietary levels of ARA fed to the high-ARA group were chosen to provoke the metabolism. We aimed to study the metabolic processes that could explain the effect that others have shown when dietary *n-*3:*n-*6 PUFA ratio changes. Thereby, we can point the effect to ARA and not its precursors. We used metabolomics to investigate the manifoldness of changes in response to feeding high dietary ARA levels for 17 d during the extensive growth period from the larval stage at 27 d post fertilisation (DPF) until juvenile stage (44 DPF). We found that high ARA levels contribute to a strong shift in lipid metabolism involving significant lipid mediators, which suggests an impact on physiological functions and challenges the redox environment in the fish.

## Methods

### Ethical considerations

The feeding experiment was approved by the Norwegian Animal Research Authority and was conducted according to current animal welfare regulations in Norway: FOR-1996-01-15-23. Facilities for zebrafish husbandry were optimally equipped to ensure refinement of breeding, accommodation and care. Handling and treatment of the fish ensured reduction of any possible pain, distress or lasting harm to the fish.

### Formulation and preparation of diets

The feeding experiment and diets were made as previously described in detail^(^ ^45^ ^)^. In short, we fed zebrafish a control diet with low ARA levels (ARA 0·19 % of DM), or an experimental diet with high ARA levels (ARA 2·10 % of DM), referred to as high-ARA diet. The ARA levels were chosen on the basis of previous studies on fish to avoid deficient levels in the low-ARA group^(^ ^22^ ^,^ ^46^ ^)^. Protein, mineral and vitamin blends as well as oil composition are provided in Table 1. Initially, all feed ingredients were mixed (protein, mineral and vitamin blends, and oil including fish oil, rape seed oil, flax seed oil and Cargill’s ARA-rich oil) with a solution of dissolved agar until a smooth texture was achieved. Astaxanthin was added to the agar solution before mixing with other feed ingredients. The feed paste was dried at 42°C for 72 h, ground and sieved into fractions of different feed-pellet sizes and stored at–20°C until feeding. The feeding regimen was as follows: <200 µm fed 27–43 DPF, 350 µm fed 44–57 DPF and 560 µm fed 58–90 DPF.

**Table 1:** Feed composition

Ingredients	Control (g/kg DM)	High-ARA (g/kg DM)
Protein blend*	767·9	767·9
Agar†	1·0	1·0
Fish oil‡	8·0	8·0
Rape seed oil§	48·0	20·0
Flax seed oil§	20·0	4·0
Cargill’s ARA-rich oil||	4·0	48·0
Dextrin†	46·17	46·17
Cellulose¶	19·3	19·3
Lecithin**	20·0	20·0
Mineral mix††	50·0	50·0
Vitamin mix‡‡	10·0	10·0
Methionine§§	2·5	2·5
Cyanocobalamin(1 %)||||	0·99998	0·99998
Folic acid (97 %)||||	0·0111	0·0111
Pyridoxine hydrochloride||||	0·0199	0·0199
Astaxanthin¶¶	0·3	0·3
Sucrose†	1·0	1·0
Tocopherol mix***	0·75	0·75

ARA, arachidonic acid.

* BioMar AS products: fishmeal, 5 %; krill meal, 1 %; soya protein concentrate, 6·2 %; maize, 5 %; wheat, 7·5 %; wheat gluten, 13 %; pea protein, 49·8 %; field peas, 12·5 %.

† Dissolved in 200 ml heated Milli-Q water; Sigma Aldrich Norway AS.

‡ Cod liver oil; Møllers, Axellus AS.

§ Rømer Produkt.

|| Donated by Cargill (40 % ARA; Alking Bioengineering).

¶ Sigma-Aldrich.

** Alfa Aesar.

†† Merck; ingredients (g/kg of diet): CaHPO_4_·2H_2_O, 30; CoCl_2_·6H_2_O, 0·007; CuSO_4_·5H_2_O, 0·02; K_2_SO_4_, 15; KI, 0·05; MgSO_4_·7H_2_O, 5; MnSO_4_·H_2_O, 0·05; NaCl, 2·873; Se-yeast, 0·2; ZnSO_4_·7H_2_O, 0·5; FeSO_4_·7H_2_O, 0·6.

‡‡ Obtained from Vilomix Norway AS, Norway; without cyanocobalamin, folic acid and pyridoxine hydrochloride (vitamin B_6_) because of the trial set up with two directions (mg/kg of diet): vitamin A, 20; vitamin D, 4; vitamin E (50 %, acetate), 200; vitamin K (50 %), 10; vitamin C (35 %, phosphate), 350; choline, 1000; ascorbic acid, 1000; thiamine hydrochloride, 15; riboflavin (80 %), 19; nicotinamide, 200; inositol, 400; calcium pantothenate, 60; biotin (2 %), 50; filler (protein blend), 6672.

§§ Sigma-Aldrich.

|||| Normin AS.

¶¶ Dissolved in the agar solution; provided as a gift from G.O. Johnsen AS.

*** Provided as a gift from BASF.

### Experimental setup

**Fig. 1:**
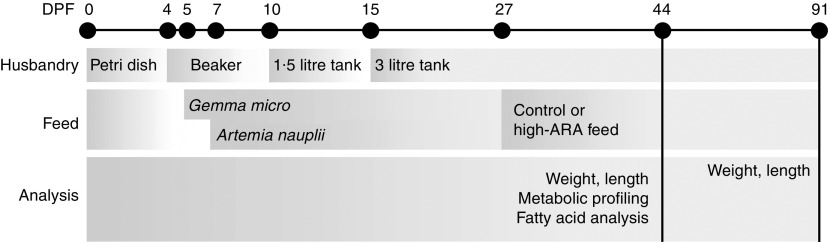
Experimental design. Zebrafish were fed *Gemma micro* and *Artemia nauplii* as start feed from 5 and 7 d post fertilisation (DPF), respectively. The experimental feeds, control or high arachidonic acid (ARA), were given to ten replicate tanks for each feed from 27 DPF onwards. Weight and length were measured at 44 and 91 DPF. Metabolic profiling and fatty acid analysis were performed at 44 DPF.

Zebrafish AB strain (*Danio rerio*) were handled and fed as previously described^(^ ^45^ ^)^. In short, zebrafish embryos were collected randomly and incubated in Petri dishes. At 4 DPF they were transferred to beakers with sixty larvae (Fig. 1). Zebrafish were fed twice a day from 5 DPF with dry feed (Gemma micro^®^; Skretting) in addition to *Artemia nauplii* (*Artemia*; Silver Star) from 7 DPF until 27 DPF. At 15 DPF, larvae were randomly transferred into 3 litre tanks in a reverse osmosis water treatment system (Aquatic Habitats recirculation system). For each diet group we assigned ten sex-mixed 3 litre tanks containing sixty fish. Each tank represents one biological replicate. Both the control and the high-ARA diet were given from 27 DPF onwards, twice a day, until 90 DPF. Fish were fed *ad libitum* from 27–43 DPF, and thereafter from 44 DPF with a restrictive diet of 7 % of the tank total biomass^(^ ^45^ ^)^. Fish were kept under steadily monitored standard conditions with 28±1°C, 14 h light–10 h dark period, conductivity of 500 µS and pH 7·5.

### Sampling and growth measures

Fish were deprived of food 18 h before sampling. In all, 44 and 91 DPF zebrafish were anaesthetised with 0·05 % tricaine methanesulfonate (MS-222; Metacain) before weighing, standard length measuring and sampling. Anaesthetised whole fish were snap-frozen with liquid N_2_ and stored at –80°C for fatty acid and metabolic profiling.

### Fatty acid analysis in feed and fish

Fatty acid composition of the dried diets and 44 DPF whole fish was determined on isolated fatty acid fractions as previously described by Jordal *et al.* ^(^ ^47^ ^)^, modified by Lie & Lambertsen^(^ ^48^ ^)^ using GLC. Quantification of fatty acids was done using 19 : 0 as the accredited internal standard and integration of peak areas was done using Dionex Chromeleon (version 7.1.3.2425). Fatty acid quantification was done in whole-44-DPF-fish homogenates, where three parallels of twenty pooled individuals were made by combining two tanks.

### Metabolic profiling

Targeted metabolic analysis involving high-throughput characterisation of all detectable metabolites was performed by Metabolon^®^, Inc. in order to examine the effect of higher ARA levels on the general metabolism in 44 DPF zebrafish. Six parallels consisting of forty pooled individuals in each parallel were sent for analysis. Targeted metabolic profiling measures defined groups of characterised metabolites in a quantitative manner using internal standards. All applied methods used Waters ACQUITY Ultra HPLC and Thermo Scientific Q-Exactive high-resolution/accurate MS interfaced with a heated electrospray ionisation source and Orbitrap mass analyser operated at 35 000 mass resolution. Sample preparation, extraction and metabolite identification was as described by Skjærven *et al.* ^(^ ^45^ ^)^. Compounds were identified by comparison with library entries of purified standards by Metabolon^®^. Values of detected compounds of known identity were normalised to the Bradford protein concentration, log-transformed and described as intensity scale.

### Statistical analysis

Data visualisation and statistical significance testing of weight, length and fatty acid analysis data were performed using GraphPad Prism version 6.00. Weight and length data were analysed using a non-parametric test (Mann–Whitney test) as none of the data showed Gaussian distribution, except weight data at 91 DPF, which was analysed by an unpaired, parametric *t* test with Welch’s correction. Fatty acid levels are presented as mean values and standard deviations and tested with an unpaired *t* test to reveal significant differences between the feed groups. Statistical significance was generally accepted at *P*<0·05.

For metabolic profiling, each value got rescaled to set the median=1, and got described as scaled intensity in tables and figures. Missing values in one sample were assumed to be below the detection limit and were imputed with the minimum value from other samples for subsequent statistical analysis. Welch’s two-sample *t* test (ArrayStudio; Omicsoft) was used on log-transformed data to identify significant (*P*<0·05) metabolites with pairwise comparison between the two feed groups. Calculation of the false discovery rate (*q* value) took into account multiple comparisons that occur in metabolic-based studies by using a cut-off point (*q*≤0·05) for indication of high confidence in a result. Relative fold changes, termed as mean ratios (MR), were calculated from high-ARA to control group using group averages of the scaled intensity values. Scaled data are presented in the online Supplementary Table S1 as a pathway heat-map including group averages of the scaled intensity values, MR, *P* and *q* values from statistical testing. Data visualisation was done using GraphPad Prism. The MetaboLync Cytoscape Plugin was used to calculate sub-pathway enrichment. Pathway enrichment scores determine the number of statistically significant regulated compounds (*k*) relative to all detected compounds (*m*) in a pathway, compared with the total number of significant regulated compounds (*n*) relative to all detected compounds (*N*) in the analysis: (*k*/*m*)/(*n*/*N*).

## Results

### High dietary arachidonic acid affected weight but not length

Dietary high ARA levels had a slight effect only at 44 DPF, where the high-ARA group was significantly lighter (*P*=0·04) compared with the control group (Table 2). At this stage, both feed groups show a large weight variation. At 91 DPF we observed no differences in weight and length (Table 2).

**Table 2:** Weight and length measures† (Mean values and standard deviations)

	Weight (mg)							Length (cm)						
	Control			High-ARA				Control			High-ARA			
DPF	Mean	sd	*n* ‡	Mean	sd	*n* ‡	*P*	Mean	sd	*n* ‡	Mean	sd	*n* ‡	*P*
44	50·72	27·43	57	40·23	25·26	48	0·04*	1·29	0·28	57	1·23	0·28	48	0·27
91	265·0	99·0	49	266·4	92·3	48	0·94	2·35	0·29	49	2·35	0·28	48	0·89

ARA, arachidonic acid; DPF, days post fertilisation. Statistically significant: * *P*<0·05.

† Statistical significance analysis was done by non-parametric Mann–Whitney test, except for 91 DPF (weight) which was analysed by a parametric *t* test with Welch’s correction. 44 DPF weight and length data and 91 DPF length data do not follow a Gaussian distribution.

‡ *n* are individual fish originated from different populations (tanks) which got summarised within the feed group for subsequent statistical analysis.

### High dietary arachidonic acid affected fatty acid profiles

Feeding of experimental diets for 17 d changed the fatty acid profiles in 44 DPF zebrafish. The ratios of *n-*3:*n-*6 PUFA with control and high-ARA feed were 0·6 and 0·2, respectively (Table 3). GLC analysis revealed six times higher ARA concentrations in the high-ARA group compared with the control group, accompanied by significantly elevated levels of its elongated and desaturated products adrenate (22 : 4*n-*6) and *n-*6 DPA (22 : 5*n-*6). Fatty acid levels are presented in Table 3 (full list of analysed fatty acids is given in the online Supplementary Table S2 (feed) and Table S3 (fish)). Oleic acid (18 : 1*n-*9) and linoleic acid (18 : 2*n-*6) were the most abundant fatty acids in both feed groups. Feeding the high-ARA diet, which consisted of relatively lower amounts of rape-seed and flax-seed oil, compared with the control feed (control 6·8 %; high ARA 2·4 %), resulted in lower levels of *α*-linolenic acid (ALA; 18 : 3*n-3*) in the high-ARA group compared with the control group. Although EPA and DHA (22 : 6*n-*3) levels were balanced in the two feeds, there was a difference in EPA and DHA levels between the control and the high-ARA group. High dietary ARA levels contributed to an altered *n-*3:*n-*6 PUFA ratio of 0·6 in the control group compared with 0·2 in the high-ARA group, indicating higher *n-*6 PUFA levels and lower *n-*3 PUFA levels in the high-ARA group.

**Table 3:** Fatty acid profiles (selected) of feed and zebrafish fed for 17 d with either control or high-arachidonic acid (ARA) feed (Mean values and standard deviations)

			Zebrafish (mg fatty acid/g fish)†				
	Feed (mg fatty acid/g feed)‡		Control		High-ARA		
	Control	High-ARA	Mean	sd	Mean	sd	*P*
18 : 1*n-*9 oleic acid	43·32	27·24	18·59	4·14	12·31	0·35	0·058
18 : 2*n-*6 linoleate	31·27	26·80	10·61	2·13	8·58	0·21	0·177
18 : 3*n-*3 *α*-linolenate	17·50	6·43	4·17	0·88	1·56	0·06	0·007**
18 : 4*n-*3 stearidonate	0·25	0·25	0·16	0·05	0·07	0·01	0·035*
20 : 3*n-*6 dihomo-linolenate	0·17	1·79	0·48	0·1	0·87	0·02	0·002**
20 : 4*n-*6 ARA	1·87	20·66	1·04	0·16	5·74	0·13	<0·001***
20 : 5*n-*3 EPA	1·26	1·3	0·47	0·08	0·32	0·02	0·038*
22 : 4*n-*6 adrenate	0·05	0·14	0·06	0·01	0·35	0·0	<0·001***
22 : 5*n-*6 docosapentaenoate (*n-*6 DPA)	0·05	0·04	0·09	0·03	0·56	0·03	<0·001***
22 : 5 *n-*3 docosapentaenoate (*n-*3 DPA)	0·15	0·15	0·14	0·03	0·12	0·01	0·148
22 : 6 *n-*3 DHA	1·42	1·37	2·13	0·27	1·63	0·05	0·034*
Sum unidentified	1·27	1·55	0·61	0·1	0·61	0·01	0·996
Sum identified	121·00	118·00	52·77	10·86	47·3	1·08	0·435
Sum SFA	17·40	25·20	10·47	2·12	11·4	0·26	0·491
Sum MUFA	49·30	31·90	22·00	4·81	15·1	0·44	0·069
Sum PUFA	54·60	60·50	20·40	3·93	20·8	0·46	0·870
Sum EPA+DHA	2·68	2·67	2·60	0·35	1·94	0·06	0·033*
Sum *n*-3 PUFA	20·90	9·75	7·57	1·41	4·08	0·11	0·013*
Sum *n*-6 PUFA	33·60	50·70	12·67	2·52	16·60	0·36	0·055
*n*-3:*n*-6	0·6	0·2	0·6	0·0	0·2	0·0	

Statistically different mean values between the control and the high-ARA group were determined using unpaired *t* test using GraphPad Prism. * *P*<0·05, ** *P*<0·01, *** *P*<0·001.

† Mean is calculated for three biological replicates consisting of twenty pooled 44 DPF zebrafish/replicate.

‡ Data are expressed as the mean of two technical replicates.

### General functional characterisation of detected metabolites from metabolic profiling

**Fig. 2:**
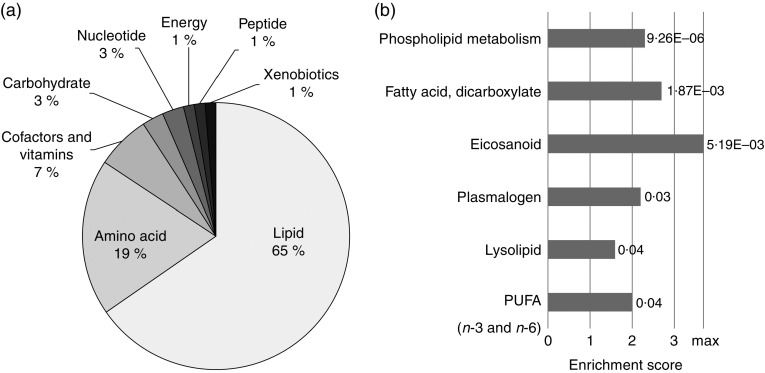
Metabolic profiling revealed complex changes in lipid metabolism. (a) Proportional clustering shows statistically different metabolites (*n* 153, *P*<0·05) affiliated to their main pathway. (b) Enrichment analysis revealed six significantly enriched sub-pathways. Scores underlie an enrichment of significantly different metabolites of total detected metabolites within a sub-pathway in relation to all significant metabolites to all detected metabolites. Graph shows statistically significant enriched (*P*<0·05) sub-pathways according to their calculated enrichment scores with indicated *P* values. The maximum achievable enrichment score is 3·7 (marked with max), if all detected metabolites in a sub-pathway are described as statistical significant different. For all sub-pathway enrichment scores see the online Supplementary Table S4.

In total, 153 out of 566 detectable compounds from metabolic profiling were statistically different between the feed groups, using Welch’s two-sample *t* test (*P*<0·05; *q*<0·05; online Supplementary Table S1). Principal component analysis shows a clear separation of the samples according to the feed groups (online Supplementary Fig. S1). The majority of metabolites affected by ARA belong to the lipid and amino acid main pathway. The lipid main pathway shows the highest count (65 %) for statistically significant metabolites among all significantly different biochemicals (Fig. 2(a)). The high-ARA group yielded a shift in lipid metabolism, especially in PUFA, complex lipids and ARA-derived eicosanoids. Moreover, a total of six sub-pathways related to lipid metabolism were enriched in the high-ARA group (Fig. 2(b)). The entire set of enriched sub-pathways is given in the online Supplementary Table S4.

### High dietary arachidonic acid affected lipid metabolism

**Fig. 3:**
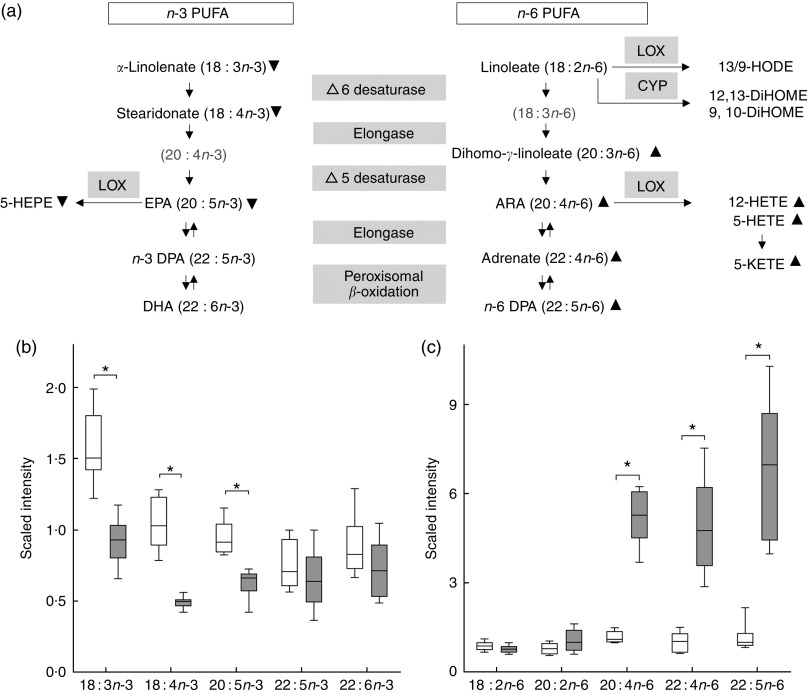
Metabolic profiling revealed changes in PUFA synthesis. (a) Metabolites illustrated in the PUFA synthesis pathway with lipoxygenase (LOX) and cytochrome P450 (CYP)-derived eicosanoid classes. 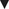, 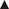, Statistically significant lower and higher metabolite levels in the high-arachidonic acid (ARA) group compared with the control group. Grey highlighted metabolites were not detected. (b) and (c) Box plots of normalised data expressed as scaled intensity of single *n*-3 and *n*-6 PUFA, respectively. 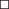, Control; 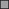, high-ARA; ARA, 20 : 4*n*-6; DHA, 22 : 6*n*-3; EPA, 20 : 5 *n*-3; *n*-3 DPA, 22 : 5 *n*-3; *n*-6 DPA, 22 : 5 *n*-6; 5-HEPE, 5-hydroxy-EPA; 5-HETE, 5-hydroxy-eicosatetraenoic acid; 12-HETE, 12-hydroxy-eicosatetraenoic acid; 5-KETE (5-oxo-ETE), 5-keto-eicosatetraenoic acid (5-oxo-eicosatetraenoic acid); 13/9-HODE, 13/9-hydroxy-octadecadienoic acid; 12,13-DiHOME, 12,13-dihydroxy-octadecenoic acid; 9,10-DiHOME, 9,10-dihydroxy-octadecenoic acid. * Significant difference (*P*<0·05) between feed groups (Welch’s two-sample *t* test).

**Fig. 4:**
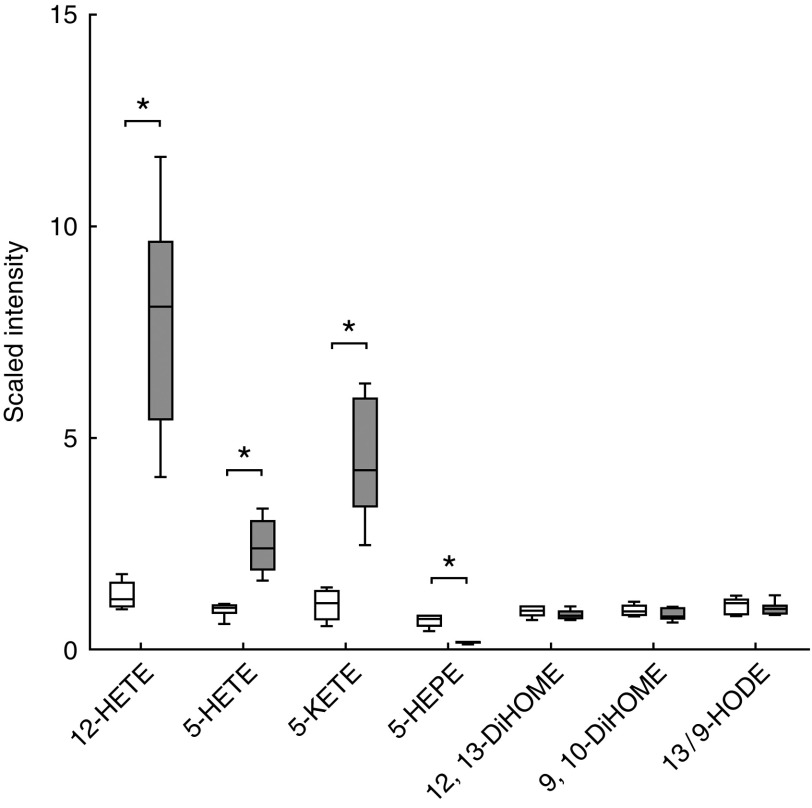
Metabolic profiling revealed changes in eicosanoids derived from linoleic acid, EPA and arachidonic acid (ARA). Box plots of normalised data are expressed as the scaled intensity of single eicosanoids. 12-HETE, 12-hydroxy-eicosatetraenoic acid; 5-HETE, 5-hydroxy-eicosatetraenoic acid; 5-KETE (5-oxo-ETE), 5-keto-eicosatetraenoic acid (5-oxo-eicosatetraenoic acid); 5-HEPE, 5-hydroxy-EPA; 12,13-DiHOME, 12,13-dihydroxy-octadecenoic acid; 9,10-DiHOME, 9,10-dihydroxy-octadecenoic acid; 13/9-HODE, 13/9-hydroxy-octadecadienoic acid; 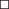, control; 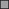, high-ARA. * Significant difference (*P*<0·05) between feed groups (Welch’s two-sample *t* test).

The high-ARA group revealed higher levels of eicosanoids, which obtained the maximum enrichment score (Fig. 2(b)). Among those, ARA-derived HETE like 5-HETE (3-fold increase), 12-HETE (6·5-fold increase) and 5-keto-eicosatetraenoic acid (5-KETE; 4-fold increase) were significantly elevated in the high-ARA group (Fig. 3(a) and 4), whereas 5-hydroxy-EPA (Fig. 3(a) and 4) was four times decreased. The latter derives from the *n-*3 PUFA EPA (20 : 5*n-*3), which was decreased, as well as its precursors ALA (in the online Supplementary Table S1 referred to as salt of ALA: *α*-linolenate; 18 : 3*n-*3) and stearidonate (18 : 4*n-*3). DHA (22 : 6*n-*3) levels detected in metabolic profiling were not different between feed groups (Fig. 3(a) and (b)), whereas GLC analysis revealed decreased DHA levels in the high-ARA group compared with the control group (Table 3). The high-ARA group exhibited decreased *n-*3 PUFA and increased *n-*6 PUFA levels compared with the control group. The high-ARA group showed a 5-fold increase in ARA (in the online Supplementary Table S1 referred to as salt of ARA: arachidonate) and adrenate and six times higher *n-*6 DPA (22 : 5*n-*6) levels compared with the control group (Fig. 3(a) and (c)).

The high-ARA group showed increased levels of arachidonoyl-containing glycerophospholipids, plasmalogens and lysophospholipids, which are arachidonoyl-containing complex lipids. Linoleoyl (18 : 2*n-*6) as well as linolenoyl-containing (18 : 3*n-*3) glycerophospholipids and lysophospholipids were decreased in the high-ARA group. One glycerophospholipid, named 1-linoleoyl-2-arachidonoyl-GPC (18 : 2/20 : 4*n-*6), was predominant among all detected phospholipids as it exhibited twelve times higher levels in the high-ARA group. Sphingolipids, especially sphingomyelins showed significantly lower levels in the high-ARA group. Dicarboxylic acids such as 2-hydroxyadipate, maleate, suberate, azelate, sebacate and pimelate, and the ketone body 3-hydroxybutyrate were significantly increased in the high-ARA group. Higher ARA levels decreased both carnitine and acetylcarnitine as well as several acylcarnitines, but also increased *cis*-4-decenoyl carnitine and stearoylcarnitine.

### High dietary arachidonic acid affected redox environment, vitamins, cofactors, carbohydrate and energy metabolism

ARA-derived endocannabinoids and monoacylglycerols like 2-arachidonoyl-glycerol and its inactive analogue 1-arachidonoyl-glycerol showed statistically higher levels in the high-ARA group. Moreover, *N*-stearoyl-taurine and *N*-palmitoyl-taurine, known as aminoacyl-endocannabinoids, had higher levels in response to higher *n-*6 PUFA levels as well. 7-hydroxy-cholesterol as well as oxidised products of amino acids and peptides like methionine-sulfoxide and *N*-acetyl-methionine-sulfoxide (oxidised products of methionine), cysteine *s*-sulfate (oxidised product of cysteine), cysteine-glutathione-disulfide and 4-hydroxy-nonenal (4-HNE)-glutathione (oxidised products of glutathione) were elevated in the high-ARA group. Cystine, the disulfide form of cysteine, was increased in the high-ARA group compared with the control group. Cysteine for glutathione synthesis was significantly decreased in the high-ARA group, whereas both GSH and GSSG decreased slightly (not significant; 0·05<*P*<0·1) in the high-ARA group. In addition, ascorbate (ascorbic acid) and its oxidised derivatives like threonate and oxalate were increased in the high-ARA group. Urate levels were three times decreased and carnosine levels were 2·5 times decreased in the high-ARA group. *δ*-tocopherol levels were decreased, whereas *α*-/*β*-/γ-tocopherol levels were unaffected in the high-ARA group. Concurrently, pyridoxate and pyridoxamine showed lower levels in the high-ARA group, whereas pyridoxal and pyridoxamine phosphate were not different. Central metabolites related to glycolysis and gluconeogenesis (glucose-6-phosphate) and the pentose phosphate way (ribose-5-phosphate) showed lower levels in the high-ARA group. Concerning the TCA cycle, *α*-ketoglutarate and succinylcarnitine were significantly decreased, and malate was slightly (not significant; *P*<0·1) decreased in the high-ARA group.

## Discussion

**Fig. 5:**
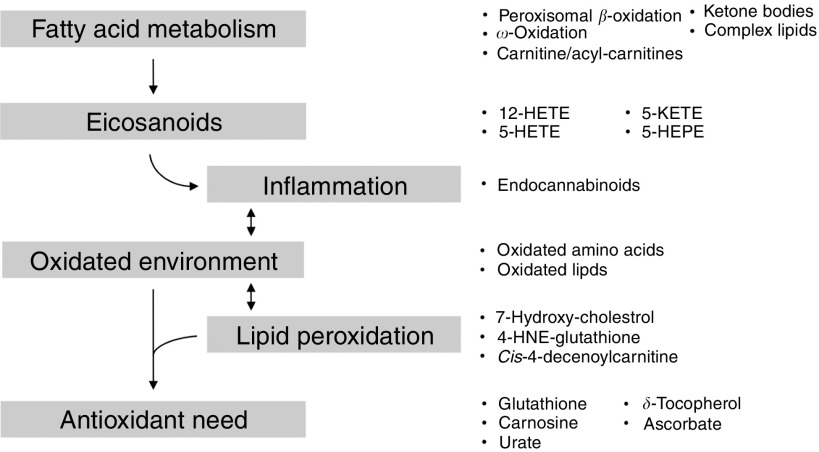
High dietary arachidonic acid changed the metabolic fingerprint in zebrafish. Changes are characterised not only by a general change in lipid profiles and eicosanoids, but also by changed metabolites indicating inflammation and lipid peroxidation and changes in the antioxidant status. Arrows indicate the suggestive physiological conditions in the fish. Metabolites to which reference is made are given on the right side. 12-HETE, 12-hydroxy-eicosatetraenoic acid; 5-KETE (5-oxo-ETE), 5-keto-eicosatetraenoic acid (5-oxo-eicosatetraenoic acid); 5-HETE, 5-hydroxy-eicosatetraenoic acid; 5-HEPE, 5-hydroxy-EPA; 4-HNE-glutathione, 4-hydroxy-nonenal-glutathione.

Metabolic profiling of zebrafish fed high-ARA levels revealed changes in complex lipids, fatty acid metabolism and immune-related eicosanoids, and suggests a challenged redox environment (Fig. 5).

### Lipid metabolism

As predicted, increasing ARA and *n-*6 PUFA levels in the feed resulted in a lower *n-*3:*n-*6 PUFA ratio in the fish. Increased ARA levels gave rise to its elongated metabolites adrenate (22 : 4*n-*6) and *n*-6 DPA (22 : 5*n-*6), which is suggestive of increased peroxisomal *β*-oxidative degradation of very-long-chain fatty acids^(^ ^49^ ^)^. *β*-Oxidation is the main catabolic pathway for long-chain fatty acids; however, when capacity is overwhelmed, minor and alternative catabolic pathways such as *ω*-oxidation may become more important^(^ ^50^ ^)^. Little research has been done on the biological significance of fatty acid *ω*-oxidation as described by Miura ^(^ ^51^ ^)^. Intermediates formed by *ω*-oxidation, such as dicarboxylic acids, accumulated in the high-ARA group, suggesting an overwhelmed *β*-oxidation. Furthermore, increased ketogenesis (3-hydroxybutyrate) and decreased carnitine and acetylcarnitine levels, as observed in the high-ARA group, are indicative of a challenged *β*-oxidation as well.

High dietary ARA levels affected fatty acid metabolism by favouring *n*-6 PUFA, but our results also highlight changes in complex lipid profiles (glycerophospholipids, plasmalogens and lysophospholipids). These changes are particularly characterised by increased levels of arachidonoyl-containing complex lipids following the high dietary ARA intake and suggesting subsequent incorporation of dietary fatty acids into complex lipids^(^ ^52^ ^)^. Changing the fatty acid composition of phospholipids can impact membrane and immune cell function in the fish. Composition of phospholipids can affect their affinity as substrates for enzymes which generate signalling molecules, and could contribute to the alteration of immune cell responsiveness as suggested by Calder & Grimble^(^ ^53^ ^)^.

### Eicosanoids

ARA can be metabolised by a variety of enzymes resulting in a complex mixture of biologically active derivatives (eicosanoids) with distinct functions^(^ ^24^ ^)^. The properties and precise roles of those eicosanoids are not fully understood in mammals, and even less is known for fish. In the present study, high dietary ARA gave rise to intermediate lipoxygenase products such as 5-HETE, 5-KETE and 12-HETE in the fish. 5-HETE can reversibly be converted to 5-KETE through an oxidation reaction, and oxidative stress could increase the conversion to 5-KETE^(^ ^24^ ^,^ ^54^ ^)^. ARA-derived eicosanoids (HETE) show a diversity in biological functions under different physiological and pathophysiological conditions^(^ ^24^ ^)^. As for PG and leukotrienes, HETE has also been described in context of inflammatory responses^(^ ^15^ ^)^. Several studies have shown that a disproportionally high intake of *n*-6 PUFA can promote inflammation, resulting in pathophysiological effects in humans^(^ ^13^ ^,^ ^14^ ^,^ ^24^ ^)^. However, it is suggested that *n-*6 PUFA and their metabolites are involved in both pro- and anti-inflammatory signalling pathways in mammals^(^ ^15^ ^,^ ^16^ ^)^. In the present study, abundant ARA could have contributed to the promotion of metabolically triggered inflammation, as suggested by others^(^ ^55^ ^–^ ^57^ ^)^. Particularly, altered fatty acid profiles can change eicosanoid production and can thereby impact physiological functions by altering the range of inflammatory and immune cell responses^(^ ^53^ ^,^ ^58^ ^)^.

In the present study, we observed elevated levels of endocannabinoids such as *N*-stearoyltaurine, *N*-palmitoyltaurine and 2-arachidonoyl-glycerol^(^ ^59^ ^)^. These endocannabinoids are understood as anti-inflammatory molecules^(^ ^60^ ^)^ induced by stress; especially, 2-arachidonoyl-glycerol plays an active role in ameliorating inflammation^(^ ^61^ ^)^. Similarly, Alvheim *et al.* ^(^ ^62^ ^)^ observed elevated levels of 2-arachidonoyl-glycerol in mice fed high levels of linoleic acid, an ARA precursor. Powell *et al.* ^(^ ^44^ ^)^ observed a decrease in chronic inflammatory response genes (C-reactive protein, serum amyloid A and vitellogenin) in zebrafish with decreasing dietary *n-3:n-*6 PUFA ratio. Whether an ARA-stimulated increase in anti-inflammatory endocannabinoids in zebrafish and mice is part of a compensatory action induced by an increase in pro-inflammatory eicosanoids is not known. Despite these divergent observations, our results suggest an inflammatory challenged metabolism in response to a low dietary *n-3:n-*6 PUFA ratio.

### Oxidative and antioxidative response

**Fig. 6:**
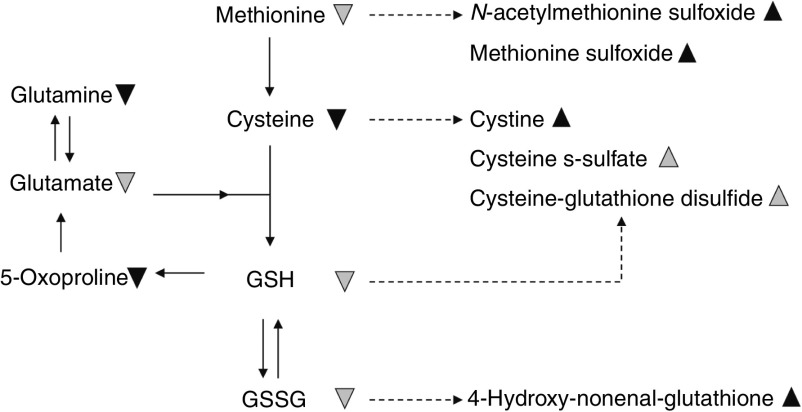
High dietary arachidonic acid (ARA) affected the redox environment, characterised by increased oxidised amino acids in zebrafish. The observed effect suggests changes in the oxidation–reduction state, indicating oxidative stress and lipid peroxidation. 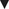, 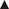, Statistically significant (*P*<0·05) lower and higher metabolite levels in the high-ARA group compared with the control group. 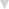, 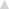, Lower and higher metabolites levels, which narrowly missed the statistical cut-off point for significance (0·05<*P*<0·1) in the high-ARA group.

An inflammatory environment is often associated with antioxidative events as a consequence of a changing oxidised environment. In the high-ARA group, we observed distinct changes in the redox environment (Fig. 6) compared with the control group. Oxidised products of lipids (7-hydroxy-cholesterol), amino acids and peptides (*cis*-4-decenoylcarnitine, methionine-sulfoxide, *N*-acetylmethionine-sulfoxide, cysteine-*S*-sulfate, cysteine-glutathione-disulfide, 4-HNE-glutathione) were increased. Especially, increasing levels of cystine and decreasing levels of cysteine reflect changes in the oxidation–reduction state in the high-ARA group. Furthermore, increased methionine-sulfoxide levels resulting from methionine oxidation can have profound functional consequences for target proteins, especially when signalling protein residues are affected^(^ ^63^ ^)^. These results indicate that increasing dietary levels of *n*-6 PUFA resulted in changes in oxidising conditions in the fish. Oxidative stress can potentiate the possibility of systemic inflammation^(^ ^7^ ^)^, which in turn affects the susceptibility for inflammation-underlying diseases^(^ ^5^ ^,^ ^6^ ^)^.

Increased levels of oxidised lipids in the high-ARA group, like 7-hydroxy-cholesterol, *cis*-4-decenoylcarnitine and 4-HNE-glutathione, have been previously connected to oxidative stress and radical-mediated lipid peroxidation in other experiments^(^ ^64^ ^–^ ^68^ ^)^. There is evidence that an increasing fatty acid unsaturation (PUFA) correlates positively with peroxidisability of lipids^(^ ^69^ ^)^. In the present study, increased levels of 4-HNE-glutathione, which results from 4-HNE detoxification^(^ ^70^ ^,^ ^71^ ^)^, suggest lipid peroxidation in the high-ARA group. 4-HNE is the major end product of reactive oxygen species-mediated peroxidation of membrane *n*-6 PUFA like ARA and linoleic acid in inflammation-related events^(^ ^64^ ^,^ ^72^ ^)^.

Formation of these oxidised products triggered an antioxidant demand to prevent increasing oxidation as indicated in the high-ARA group. Interestingly, some metabolites with known antioxidative properties such as glutathione, carnosine, *δ*-tocopherol and urate^(^ ^73^ ^)^ were decreased, whereas ascorbate^(^ ^74^ ^)^ was increased in the high-ARA group. Zebrafish, like humans, depend on dietary uptake of ascorbate^(^ ^75^ ^,^ ^76^ ^)^, which suggests an increased uptake from the feed rather than an increased endogenous synthesis of ascorbate in the high-ARA group. At the same time, decreased glutathione levels are consistent with an increasing demand for 4-HNE detoxification through glutathione. In cell-culture studies, increased conversion of ARA into HETE showed an increase in oxygen free radicals accompanied by glutathione depletion (GSH) that was leading to cellular damage^(^ ^77^ ^)^. Taken together, increasing *n*-6 PUFA availability in high-ARA fish led to an antioxidative response due to an enrichment of several oxidative products originating from enzymatic and non-enzymatic oxidation, indicating increasing lipid-peroxidation.

### Zebrafish growth

We observed a slight weight difference in juvenile zebrafish (only 44 DPF) between the feed groups. Developmental processes during larval and juvenile stages are especially sensitive to a dietary imbalance^(^ ^78^ ^)^. Higher susceptibility to dietary imbalances during larval and early juvenile stages might have contributed to the growth effect we observed in 44 DPF fish that disappeared later at the adult stage. Although we do not know the mechanisms behind the growth recovery, compensatory growth has been demonstrated in fish following food deprivation^(^ ^79^ ^)^. Lie *et al.* ^(^ ^23^ ^)^ and de Vrieze *et al.* ^(^ ^22^ ^)^ observed no growth effect on cod larvae and zebrafish, respectively, in response to high dietary ARA levels. Boglino *et al*.^(^ ^46^ ^)^ showed that both too-high and too-low *n*-6 PUFA levels did affect the growth of Senegalese sole larvae. Meinelt *et al.* ^(^ ^42^ ^)^ showed a positive correlation of higher dietary *n*-6 PUFA levels with growth in zebrafish, just as other animal and human studies show an association between higher *n*-6 PUFA intake and weight gain^(^ ^46^ ^,^ ^80^ ^)^. Different nutritional composition of the diets, like single fatty acid balance, magnitude of the dietary *n*-3:*n*-6 PUFA ratio, minerals and vitamins, might explain the difference in growth.

### Conclusions

We have shown that high dietary ARA levels dramatically affect *n*-3:*n*-6 PUFA profiles, especially ARA-derived eicosanoids, which can greatly impact physiologic outcomes in the fish. Lipid peroxidation and an oxidised and pro-inflammatory environment, as implicated by our results, may result from both high *n*-6 PUFA availability and a shift in ARA-derived pro- and anti-inflammatory eicosanoids in zebrafish. The effect was characterised not only by a general change in lipid profiles and eicosanoids, but also by changed metabolites, indicating lipid peroxidation, oxidation of amino acids and changes in antioxidant status. However, the link between a low dietary *n-3:n-*6 PUFA ratio, elevated eicosanoid and endocannabinoid levels, and the regulation of the redox and immune system needs to be further studied, which is required to elucidate the underlying mechanisms. To our knowledge, the present study is the first to use metabolomics to reveal the metabolic fingerprint of high dietary ARA levels in teleosts. Previous studies using metabolic profiling have focused on the involvement of ARA pathways in vascular endothelial cells and CVD^(^ ^81^ ^–^ ^83^ ^)^. We find that zebrafish can be a useful vertebrate model to study the impact of nutrients on the manifoldness of the metabolic fingerprint. Our results from juvenile zebrafish highlight the metabolic fingerprint shaped by a specific diet.

## Supplementary material

For supplementary material accompanying this paper visit http://dx.doi.org/10.1017/S0007114517000903. click here to view supplementary material
