# Supplementary material for: High dietary arachidonic acid levels induce changes in complex lipids and immune-related eicosanoids and increase levels of oxidised metabolites in zebrafish (Danio rerio)
Source: Br J Nutr. 2017 May 9;117(8):1075–85. doi: 10.1017/S0007114517000903 (PMC5481881; doi:10.1017/S0007114517000903)
Supplement: Supplementary file 1 [file S0007114517000903sup.zip › S0007114517000903sup001/S0007114517000903sup002.docx]

**Supplementary material for:**

**High dietary arachidonic acid levels induce changes in complex lipids, immune-related eicosanoids and increase levels of oxidized metabolites in zebrafish (*Danio rerio*).**

**Anne-Catrin Adam^1^*, Kai Kristoffer Lie^1^, Mari Moren^2^, Kaja Helvik Skjærven^1^**

^1^ National Institute of Nutrition and Seafood Research (NIFES), PO Box 2029 Nordnes, NO-5817 Bergen, Norway

^2^ NIFES, Bergen, Norway; current address: Nofima AS, PO Box 1425 Oasen, NO-5828 Bergen, Norway

*** Corresponding author:**

Anne-Catrin Adam

National Institute of Nutrition and Seafood Research (NIFES)

PO Box 2029 Nordnes, 5817 Bergen, Norway

Email address: aad@nifes.no

Telephone: +4748341656

Fax: +4755905299

**Table S2.** Fatty acid profile (mg fatty acid/g feed) of control and high ARA feed given to juvenile zebrafish from 27 until 90 DPF measured by GLC

| Fatty acids  (mg fatty acid/g feed) | Control feed ^a^ | High ARA feed ^a^ |
| --- | --- | --- |
| 06 : 0 | 0·01 | 0·01 |
| 08 : 0 | 0·01 | 0·01 |
| 10 : 0 | 0·03 | 0·04 |
| 12 : 0 | 0·01 | 0·01 |
| 14 : 0 | 0·69 | 0·84 |
| 14 : 1*n-*9 | 0·02 | 0·02 |
| 15 : 0 | 0·13 | 0·17 |
| 16 : 0 | 11·14 | 11·84 |
| 16 : 1*n-*9 | 0·09 | 0·06 |
| 16 : 1*n-*7 | 0·97 | 0·95 |
| 17 : 0 | 0·11 | 0·16 |
| 16 : 2*n-*4 | 0·07 | 0·07 |
| 18 : 0 | 3·78 | 5·24 |
| 16 : 3*n-*3 | 0·08 | 0·08 |
| 18 : 1*n-*11 | 0·16 | 0·01 |
| 18 : 1*n-*9 | 43·32 | 27·24 |
| 18 : 1*n-*7 | 2·40 | 1·45 |
| 16 : 4*n-*3 | 0·08 | 0·05 |
| 18 : 2*n-*6 (LA) | 31·27 | 26·80 |
| 18 : 3*n-*6 | 0·10 | 1·06 |
| 20 : 0 | 0·58 | 0·67 |
| 18 : 3*n-*3 (ALA) | 17·50 | 6·43 |
| 20 : 1*n-*11 | 0·08 | 0·08 |
| 20 : 1*n-*9 | 1·52 | 1·28 |
| 20 : 1*n-*7 | 0·03 | 0·03 |
| 18 : 4*n-*3 | 0·25 | 0·25 |
| 20 : 2*n-*6 | 0·11 | 0·22 |
| 20 : 3*n-*9 | 0·01 | 0·01 |
| 20 : 3*n-*6 | 0·17 | 1·79 |
| 22 : 0 | 0·37 | 1·61 |
| 20 : 3*n-*3 | <0·01 | <0·01 |
| 20 : 4*n-*6 (ARA) | 1·87 | 20·66 |
| 22 : 1*n-*11 | 0·53 | 0·56 |
| 22 : 1*n-*9 | 0·12 | 0·04 |
| 20 : 4*n-*3 | 0·06 | 0·07 |
| 20 : 5*n-*3 (EPA) | 1·26 | 1·30 |
| 24 : 0 | 0·58 | 4·62 |
| 22 : 4*n-*6 | 0·05 | 0·14 |
| 21 : 5*n-*3 | 0·04 | 0·02 |
| 24 : 1*n-*9 | 0·09 | 0·14 |
| 22 : 5*n-*6 | 0·05 | 0·04 |
| 22 : 5*n-*3 | 0·15 | 0·15 |
| 22 : 6*n-*3 (DHA) | 1·42 | 1·37 |
| 24 : 5*n-*3 | 0·03 | 0·02 |
| 24 : 6*n-*3 | 0·01 | 0·01 |
| Sum unidentified | 1·27 | 1·55 |
| Sum identified | 121 | 118 |
| Sum fatty acids | 123 | 119 |
| Sum saturated | 17·4 | 25·2 |
| Sum 16 : 1 | 1·06 | 1·02 |
| Sum 18 : 1 | 45·9 | 28·7 |
| Sum 20 : 1 | 1·63 | 1·40 |
| Sum 22 : 1 | 0·643 | 0·596 |
| Sum monounsaturated | 49·3 | 31·9 |
| Sum EPA + DHA | 2·68 | 2·67 |
| Sum *n*-3 PUFA | 20·9 | 9·75 |
| Sum *n*-6 PUFA | 33·6 | 50·7 |
| Sum polyunsaturated | 54·6 | 60·5 |
| *(n*-3) / (*n*-6) | 0·6 | 0·2 |

^a^ Data expressed as mean from two technical replicates.

**Table S3.** Fatty acid profile in 44 DPF zebrafish measured by GLC

| Fatty acid  (mg fatty acid/g fish) | Control | |  | High ARA | |  | Control vs.  High ARA |
| --- | --- | --- | --- | --- | --- | --- | --- |
|  | Mean | SD |  | Mean | SD |  | *P* |
| 06 : 0 |  |  |  |  |  |  |  |
| 08 : 0 |  |  |  |  |  |  |  |
| 10 : 0 |  |  |  |  |  |  |  |
| 12 : 0 | 0·017 | 0·006 |  | 0·020 | 0·000 |  | 0·373901 |
| 14 : 0 | 0·343 | 0·080 |  | 0·383 | 0·015 |  | 0·443939 |
| 14 : 1*n-*9 | 0·027 | 0·006 |  | 0·023 | 0·006 |  | 0·518519 |
| 15 : 0 | 0·140 | 0·026 |  | 0·140 | 0·000 |  | 1 |
| 16 : 0 | 7·323 | 1·528 |  | 7·370 | 0·205 |  | 0·96069 |
| 16 : 1*n-*9 | 0·313 | 0·057 |  | 0·260 | 0·010 |  | 0·184849 |
| 16 : 1*n-*7 | 0·897 | 0·196 |  | 0·813 | 0·025 |  | 0·504673 |
| 17 : 0 | 0·230 | 0·046 |  | 0·233 | 0·006 |  | 0·906554 |
| 16 : 2*n-*4 | 0·133 | 0·031 |  | 0·113 | 0·006 |  | 0·327635 |
| 18 : 0 | 2·153 | 0·392 |  | 2·580 | 0·060 |  | 0·13591 |
| 16 : 3*n-*3 | 0·080 | 0·020 |  | 0·073 | 0·006 |  | 0·608653 |
| 18 : 1*n-*11 | 0·060 | 0·000 |  | 0·040 | 0·000 |  |  |
| 18 : 1*n-*9 | 18·593 | 4·136 |  | 12·310 | 0·349 |  | 0·0586939 |
| 18 : 1*n-*7 | 1·297 | 0·252 |  | 0·960 | 0·010 |  | 0·0815588 |
| 16 : 4*n-*3 |  |  |  |  |  |  |  |
| 18 : 2*n-*6 (LA) | 10·610 | 2·132 |  | 8·583 | 0·205 |  | 0·176561 |
| 18 : 3*n-*6 | 0·230 | 0·066 |  | 0·347 | 0·012 |  | * 0·0385952 |
| 20 : 0 | 0·103 | 0·015 |  | 0·103 | 0·006 |  | 1 |
| 18 : 3*n-*3 (ALA) | 4·167 | 0·884 |  | 1·557 | 0·055 |  | ** 0·00695511 |
| 20 : 1*n-*11 | 0·097 | 0·025 |  | 0·080 | 0·010 |  | 0·34649 |
| 20 : 1*n-*9 | 0·500 | 0·105 |  | 0·380 | 0·017 |  | 0·12343 |
| 20 : 1*n-*7 |  |  |  |  |  |  |  |
| 18 : 4*n-*3 | 0·163 | 0·050 |  | 0·070 | 0·010 |  | * 0·0345059 |
| 20 : 2*n-*6 | 0·167 | 0·032 |  | 0·170 | 0·010 |  | 0·872158 |
| 20 : 3*n-*9 |  |  |  |  |  |  |  |
| 20 : 3*n-*6 | 0·480 | 0·095 |  | 0·870 | 0·020 |  | ** 0·00227562 |
| 22 : 0 | 0·083 | 0·012 |  | 0·207 | 0·006 |  | *** 7·81238E-05 |
| 20 : 3*n-*3 | 0·010 | 0·000 |  | 0·077 | 0·006 |  | *** 0·000584356 |
| 20 : 4*n-*6 (ARA) | 1·043 | 0·155 |  | 5·743 | 0·127 |  | *** 2·19531E-06 |
| 22 : 1*n-*11 | 0·123 | 0·021 |  | 0·193 | 0·015 |  | ** 0·00933784 |
| 22 : 1*n-*9 | 0·017 | 0·006 |  | 0·013 | 0·006 |  | 0·518519 |
| 20 : 4*n-*3 | 0·230 | 0·050 |  | 0·100 | 0·000 |  | * 0·010795 |
| 20 : 5*n-*3 (EPA) | 0·467 | 0·081 |  | 0·320 | 0·020 |  | * 0·0379966 |
| 24 : 0 | 0·077 | 0·015 |  | 0·347 | 0·015 |  | *** 2·69348E-05 |
| 22 : 4*n-*6 | 0·060 | 0·010 |  | 0·350 | 0·000 |  | *** 9·40091E-07 |
| 21 : 5*n-*3 | 0·027 | 0·006 |  | 0·040 | 0·000 |  | * 0·0161301 |
| 24 : 1*n-*9 | 0·043 | 0·006 |  | 0·040 | 0·000 |  | 0·373901 |
| 22 : 5*n-*6 | 0·087 | 0·025 |  | 0·557 | 0·031 |  | *** 3·30109E-05 |
| 22 : 5*n-*3 | 0·143 | 0·025 |  | 0·117 | 0·006 |  | 0·148148 |
| 22 : 6*n-*3 (DHA) | 2·127 | 0·269 |  | 1·627 | 0·049 |  | * 0·0338107 |
| 24 : 5*n-*3 | 0·047 | 0·006 |  | 0·033 | 0·006 |  | * 0·0474207 |
| 24 : 6*n-*3 | 0·113 | 0·021 |  | 0·063 | 0·006 |  | * 0·016011 |
| Sum unidentified | 0·606 | 0·099 |  | 0·607 | 0·012 |  | 0·995674 |
| Sum identified | 52·767 | 10·862 |  | 47·300 | 1·082 |  | 0·434632 |
| Sum fatty acids | 53·400 | 10·967 |  | 47·900 | 1·082 |  | 0·436115 |
| Sum saturated | 10·467 | 2·120 |  | 11·400 | 0·265 |  | 0·491322 |
| Sum 16 : 1 | 1·211 | 0·260 |  | 1·070 | 0·010 |  | 0·401807 |
| Sum 18 : 1 | 19·933 | 4·406 |  | 13·333 | 0·321 |  | 0·0608359 |
| Sum 20 : 1 | 0·596 | 0·133 |  | 0·463 | 0·032 |  | 0·169408 |
| Sum 22 : 1 | 0·145 | 0·028 |  | 0·210 | 0·017 |  | * 0·0275717 |
| Sum monounsaturated | 22·000 | 4·812 |  | 15·100 | 0·436 |  | 0·0687074 |
| Sum EPA + DHA | 2·597 | 0·350 |  | 1·943 | 0·064 |  | * 0·0334292 |
| Sum *n*-3 PUFA | 7·570 | 1·412 |  | 4·080 | 0·111 |  | * 0·0129635 |
| Sum *n*-6 PUFA | 12·667 | 2·517 |  | 16·600 | 0·361 |  | 0·0552434 |
| Sum polyunsaturated | 20·400 | 3·934 |  | 20·800 | 0·458 |  | 0·869649 |
| *(n*-3) / (*n*-6) | 0·600 | 0·000 |  | 0·200 | 0·000 |  |  |

* *P*<0·05, ** *P*<0·01, *** *P*<0·001. Statistical different mean values were determined using unpaired *t-*test using GraphPad Prism. Fatty acid levels under detection limit (<0·01 mg/g homogenate) were not stated (empty data). Means are calculated of 3 biological replicates consisting of 20 pooled 44 DPF zebrafish per replicate.

**Table S4.** Significant different detected metabolites from metabolic screening between control and high ARA group according to their sub-pathway affiliation and their enrichment score from enrichment analysis

| Sub-pathway | Enrichment score | *P* | Significant (k) | Detected  (m) | Significant metabolites |
| --- | --- | --- | --- | --- | --- |
| Bacterial/fungal | 3·7 | 0·27 | 1 | 1 | tartronate (hydroxymalonate) |
| Eicosanoid | **3·7** | **5·19E-03** | **4** | **4** | **12-HETE, 5-HEPE, 5-HETE, 5-KETE** |
| Fatty acid metabolism  (acyl choline) | 3·7 | 0·27 | 1 | 1 | palmitoylcholine |
| Fatty acid, amino | 3·7 | 0·27 | 1 | 1 | 2-aminoheptanoate |
| Fatty acid, keto | 3·7 | 0·27 | 1 | 1 | 1-dihomo-linoleoylglycerol (20:2) |
| Hemoglobin and porphyrin metabolism | 3·7 | 0·27 | 1 | 1 | heme |
| Ketone bodies | 3·7 | 0·27 | 1 | 1 | 3-hydroxybutyrate (BHBA) |
| Phosphatidylserine (PS) | 3·7 | 0·27 | 1 | 1 | 1-stearoyl-2-arachidonoyl-GPS (18:0/20:4) |
| Thiamine metabolism | 3·7 | 0·27 | 1 | 1 | 5-(2-Hydroxyethyl)-4-methylthiazole |
| Ascorbate and aldarate metabolism | 2·8 | 0·06 | 3 | 4 | ascorbate (Vitamin C), oxalate (ethanedioate), threonate |
| Fatty acid, dicarboxylate | **2·7** | **1·87E-03** | **8** | **11** | **2-hydroxyadipate, 2-hydroxyglutarate, azelate (nonanedioate), maleate, pimelate (heptanedioate), sebacate (decanedioate), suberate (octanedioate), undecanedioate** |
| Phospholipid metabolism | **2·3** | **9·26E-06** | **22** | **36** | **1,2-dilinoleoyl-GPC (18:2/18:2), 1,2-dioleoyl-GPC (18:1/18:1)*, 1,2-distearoyl-GPC (18:0/18:0), 1-linoleoyl-2-arachidonoyl-GPC (18:2/20:4*n*-6)*, 1-oleoyl-2-linoleoyl-GPC (18:1/18:2)*, 1-oleoyl-2-linoleoyl-GPE (18:1/18:2)*, 1-palmitoleoyl-2-linoleoyl-GPC (16:1/18:2)*, 1-palmitoyl-2-arachidonoyl-GPC (16:0/20:4), 1-palmitoyl-2-arachidonoyl-GPE (16:0/20:4)*, 1-palmitoyl-2-linolenoyl-GPC (16:0/18:3)*, 1-palmitoyl-2-linoleoyl-GPC (16:0/18:2), 1-palmitoyl-2-linoleoyl-GPE (16:0/18:2), 1-palmitoyl-2-linoleoyl-GPS (16:0/18:2), 1-palmitoyl-2-oleoyl-GPC (16:0/18:1), 1-palmitoyl-2-oleoyl-GPE (16:0/18:1), 1-stearoyl-2-arachidonoyl-GPC (18:0/20:4), 1-stearoyl-2-arachidonoyl-GPE (18:0/20:4), 1-stearoyl-2-arachidonoyl-GPI (18:0/20:4), 1-stearoyl-2-linoleoyl-GPC (18:0/18:2)*, 1-stearoyl-2-linoleoyl-GPE (18:0/18:2)*, phosphoethanolamine, trimethylamine N-oxide** |
| Plasmalogen | **2·2** | **0·03** | **6** | **10** | **1-(1-enyl-palmitoyl)-2-arachidonoyl-GPC (P-16:0/20:4)*, 1-(1-enyl-palmitoyl)-2-linoleoyl-GPC (P-16:0/18:2)*, 1-(1-enyl-palmitoyl)-2-linoleoyl-GPE (P-16:0/18:2)*, 1-(1-enyl-palmitoyl)-2-palmitoyl-GPC (P-16:0/16:0)*, 1-(1-enyl-stearoyl)-2-arachidonoyl-GPE (P-18:0/20:4)*, 1-(1-enyl-stearoyl)-2-linoleoyl-GPE (P-18:0/18:2)*** |
| Polyunsaturated fatty acid  (*n-*3 and *n-*6 PUFA) | **2** | **0·04** | **7** | **13** | **adrenate (22:4*n-*6), arachidonate (20:4*n-*6), dihomo-linolenate (20:3*n-*3 or *n-*6), docosapentaenoate (*n*-6 DPA; 22:5*n-*6), eicosapentaenoate (EPA; 20:5*n*-3), linolenate [alpha or gamma; (18:3*n*-3 or *n-*6)], stearidonate (18:4*n*-3)** |
| Carnitine metabolism | 1·8 | 0·47 | 1 | 2 | carnitine |
| Diacylglycerol | 1·8 | 0·3 | 2 | 4 | 1-oleoyl-2-linoleoyl-glycerol (18:1/18:2), 1-oleoyl-3-linoleoyl-glycerol (18:1/18:2) |
| Dipeptide derivative | 1·8 | 0·47 | 1 | 2 | carnosine |
| Fatty acid metabolism  (also BCAA metabolism) | 1·8 | 0·47 | 1 | 2 | butyrylcarnitine |
| Fatty acid, monohydroxy | 1·8 | 0·3 | 2 | 4 | 2-hydroxypalmitate, 2-hydroxystearate |
| Medium chain fatty acid | 1·8 | 0·47 | 1 | 2 | 10-undecenoate (11:1*n*-1) |
| Pterin metabolism | 1·8 | 0·47 | 1 | 2 | pterin |
| Vitamin B6 metabolism | 1·8 | 0·3 | 2 | 4 | pyridoxamine, pyridoxate |
| Lysolipid | **1·6** | **0·04** | **13** | **30** | **1-arachidonoyl-GPA (20:4), 1-arachidonoyl-GPC (20:4*n*-6)*, 1-arachidonoyl-GPE (20:4*n*-6)*, 1-arachidonoyl-GPI (20:4)*, 1-lignoceroyl-GPC (24:0), 1-linolenoyl-GPC (18:3)*, 1-linoleoyl-GPC (18:2), 1-linoleoyl-GPE (18:2)*, 1-linoleoyl-GPS (18:2)*, 1-oleoyl-GPC (18:1), 1-oleoyl-GPE (18:1), 1-palmitoyl-GPI (16:0)*, 1-stearoyl-GPA (18:0)** |
| Sphingolipid metabolism | 1·5 | 0·11 | 9 | 22 | lactosyl-N-palmitoyl-sphingosine, N-palmitoyl-sphingosine (d18:1/16:0), sphingomyelin (d18:1/20:0, d16:1/22:0)*, sphingomyelin (d18:1/21:0, d17:1/22:0, d16:1/23:0)*, sphingomyelin (d18:1/22:1, d18:2/22:0, d16:1/24:1)*, sphingomyelin (d18:1/24:1, d18:2/24:0)*, sphingomyelin (d18:2/23:0, d18:1/23:1, d17:1/24:1)*, sphingomyelin (d18:2/24:1, d18:1/24:2)*, tricosanoyl sphingomyelin (d18:1/23:0)* |
| Sterol | 1·5 | 0·41 | 2 | 5 | 7-hydroxycholesterol (alpha or beta), campesterol |
| Glutathione metabolism | 1·4 | 0·37 | 3 | 8 | 4-hydroxy-nonenal-glutathione, 5-oxoproline, S-methylglutathione |
| Long chain fatty acid | 1·3 | 0·32 | 5 | 14 | arachidate (20:0), erucate (22:1*n*-9), myristate (14:0), nonadecanoate (19:0), stearate (18:0) |
| Glycine, serine and threonine metabolism | 1·2 | 0·46 | 3 | 9 | dimethylglycine, N-acetylthreonine, threonine |
| Leucine, isoleucine and valine metabolism | 1·2 | 0·33 | 7 | 21 | 2-methylbutyrylcarnitine (C5), alpha-hydroxyisovalerate, beta-hydroxyisovalerate, beta-hydroxyisovaleroylcarnitine, isovalerate, N-acetylvaline, tiglylcarnitine |
| Riboflavin metabolism | 1·2 | 0·61 | 1 | 3 | flavin mononucleotide (FMN) |
| Tryptophan metabolism | 1·2 | 0·51 | 2 | 6 | 5-hydroxyindoleacetate, C-glycosyltryptophan |
| Fatty acid metabolism  (acyl carnitine) | 1·1 | 0·48 | 4 | 13 | acetylcarnitine, cis-4-decenoyl carnitine, myristoleoylcarnitine*, stearoylcarnitine |
| Methionine, cysteine, SAM and taurine metabolism | 1 | 0·56 | 5 | 18 | cysteine, cystine, hypotaurine, methionine sulfoxide, N-acetylmethionine sulfoxide |
| Monoacylglycerol | 1 | 0·62 | 5 | 19 | 1-arachidonylglycerol (20:4), 1-dihomo-linolenylglycerol (20:3), 2-arachidonoylglycerol (20:4), 2-myristoylglycerol (14:0), 2-palmitoylglycerol (16:0) |
| Endocannabinoid | 0·9 | 0·68 | 2 | 8 | N-palmitoyltaurine, N-stearoyltaurine |
| Lysine metabolism | 0·9 | 0·73 | 3 | 13 | 5-aminovalerate, glutarate (pentanedioate), saccharopine |
| Lysoplasmalogen | 0·9 | 0·72 | 1 | 4 | 1-(1-enyl-oleoyl)-GPE (P-18:1)* |
| Pentose phosphate pathway | 0·9 | 0·72 | 1 | 4 | ribose 5-phosphate |
| Phenylalanine and tyrosine metabolism | 0·9 | 0·73 | 3 | 13 | 3-methoxytyrosine, 4-hydroxyphenylpyruvate, N-acetylphenylalanine |
| Tocopherol metabolism | 0·9 | 0·72 | 1 | 4 | delta-tocopherol |
| TCA cycle | 0·8 | 0·75 | 2 | 9 | alpha-ketoglutarate, succinylcarnitine |
| Glutamate metabolism | 0·7 | 0·84 | 2 | 11 | 4-hydroxyglutamate, glutamine |
| Purine metabolism, (hypo)xanthine/inosine containing | 0·7 | 0·84 | 2 | 11 | allantoic acid, urate |
| Fructose, mannose and galactose metabolism | 0·6 | 0·85 | 1 | 6 | mannose-6-phosphate |
| Aminosugar metabolism | 0·5 | 0·92 | 1 | 8 | glucosamine-6-phosphate |
| Food component/plant | 0·4 | 0·94 | 1 | 9 | ergothioneine |
| Glycolysis, gluconeogenesis, and pyruvate metabolism | 0·4 | 0·96 | 1 | 10 | glucose 6-phosphate |
| Purine metabolism, guanine containing | 0·4 | 0·94 | 1 | 9 | guanosine 3'-monophosphate (3'-GMP) |
| Gamma-glutamyl amino acid | 0·3 | 0·98 | 1 | 12 | gamma-glutamylglutamine |
| Purine metabolism, adenine containing | 0·3 | 0·99 | 1 | 14 | adenosine 3'-monophosphate (3'-AMP) |
| Urea cycle; arginine and proline metabolism | 0·2 | 1 | 1 | 17 | pro-hydroxy-pro |
| Advanced glycation end-product | 1 | 1 | 0 | 1 |  |
| Alanine and aspartate metabolism | 1 | 1 | 0 | 6 |  |
| Biotin metabolism | 1 | 1 | 0 | 1 |  |
| Chemical | 1 | 1 | 0 | 5 |  |
| Creatine metabolism | 1 | 1 | 0 | 4 |  |
| Dipeptide | 1 | 1 | 0 | 13 |  |
| Drug | 1 | 1 | 0 | 1 |  |
| Fatty acid synthesis | 1 | 1 | 0 | 1 |  |
| Fatty acid, amide | 1 | 1 | 0 | 3 |  |
| Fatty acid, branched | 1 | 1 | 0 | 3 |  |
| Fatty acid, dihydroxy | 1 | 1 | 0 | 2 |  |
| Glycerolipid metabolism | 1 | 1 | 0 | 3 |  |
| Glycogen metabolism | 1 | 1 | 0 | 5 |  |
| Guanidino and acetamido metabolism | 1 | 1 | 0 | 2 |  |
| Histidine metabolism | 1 | 1 | 0 | 13 |  |
| Inositol metabolism | 1 | 1 | 0 | 3 |  |
| Mevalonate metabolism | 1 | 1 | 0 | 1 |  |
| Nicotinate and nicotinamide metabolism | 1 | 1 | 0 | 7 |  |
| Nucleotide sugar | 1 | 1 | 0 | 6 |  |
| Oxidative phosphorylation | 1 | 1 | 0 | 2 |  |
| Pantothenate and CoA metabolism | 1 | 1 | 0 | 1 |  |
| Pentose metabolism | 1 | 1 | 0 | 5 |  |
| Polyamine metabolism | 1 | 1 | 0 | 5 |  |
| Primary bile acid metabolism | 1 | 1 | 0 | 4 |  |
| Purine and pyrimidine metabolism | 1 | 1 | 0 | 1 |  |
| Pyrimidine metabolism, cytidine containing | 1 | 1 | 0 | 8 |  |
| Pyrimidine metabolism, orotate containing | 1 | 1 | 0 | 2 |  |
| Pyrimidine metabolism, thymine containing | 1 | 1 | 0 | 5 |  |
| Pyrimidine metabolism, uracil containing | 1 | 1 | 0 | 11 |  |
| Secondary bile acid metabolism | 1 | 1 | 0 | 1 |  |
| Steroid | 1 | 1 | 0 | 1 |  |
| Tetrahydrobiopterin metabolism | 1 | 1 | 0 | 2 |  |
| SUM |  |  | 153 | 566 |  |

* Indicates compounds that have not been officially confirmed based on a standard, but we are confident in its identity.

Data is shown according to the pathway enrichment scores in descending order. Bold data represents significant enriched sub-pathways (*P*<0·05). Enrichment scores determine the number of statistically significant regulated compounds (k) relative to all detected compounds (m) in a pathway, compared with the total number of significant regulated compounds (n=153) relative to all detected compounds (N=566) in the analysis: (k/m)/(n/N). The maximum achievable enrichment score is 3·7, if all detected metabolites in a sub-pathway are described as statistical significant different (*P*<0·05).

**
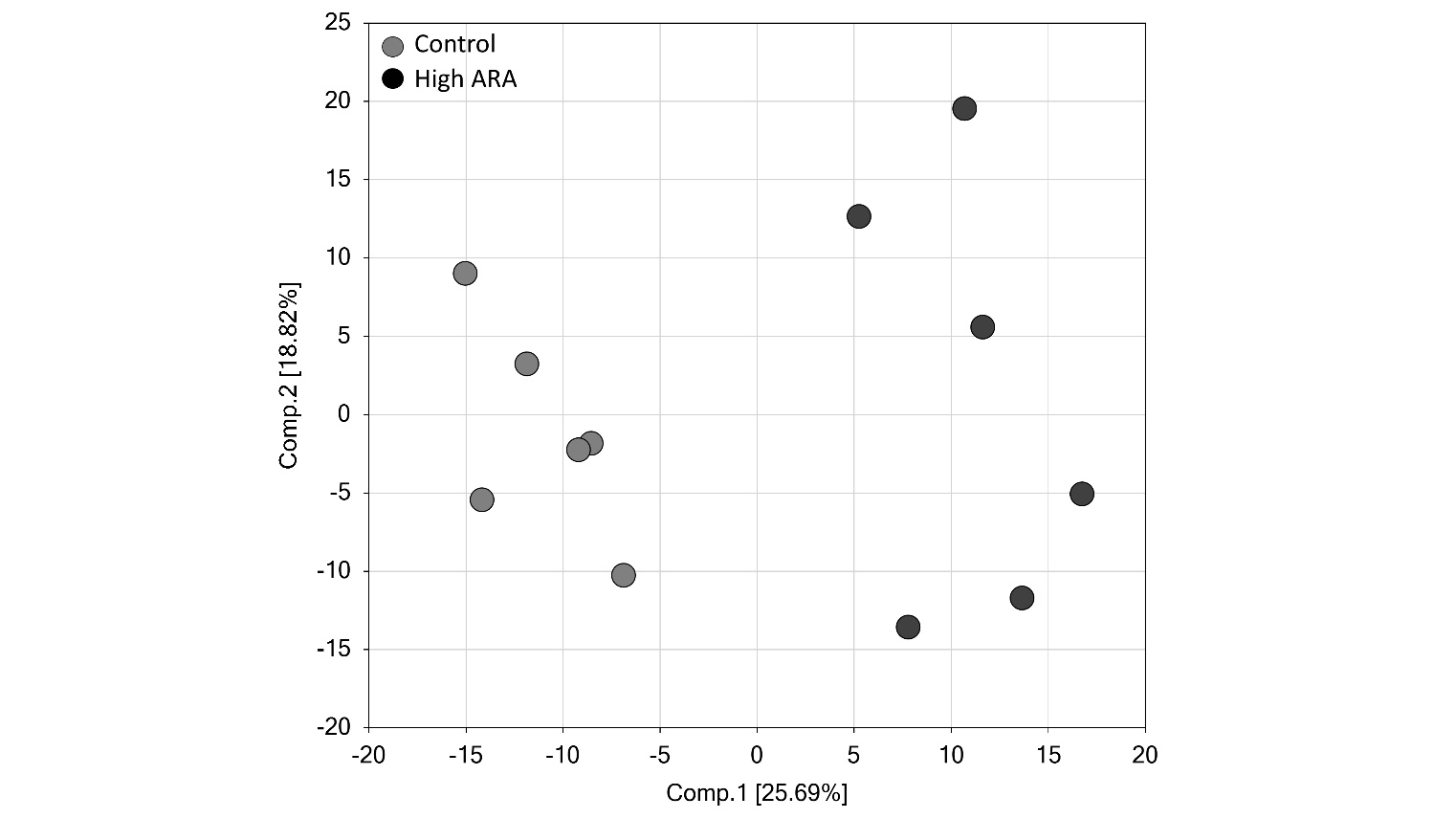
**

**Fig. S1.** Principle component analysis of metabolic profiling from 44 DPF zebrafish fed either control or high ARA feed from 27 DPF until sampling.
